# Alendronate release from calcium phosphate cement for bone regeneration in osteoporotic conditions

**DOI:** 10.1038/s41598-018-33692-5

**Published:** 2018-10-18

**Authors:** Claire I. A. van Houdt, Paulo R. Gabbai-Armelin, Paula M. Lopez-Perez, Dietmar J. O. Ulrich, John A. Jansen, Ana Claudia M. Renno, Jeroen J. J. P. van den Beucken

**Affiliations:** 1 Radboudumc, Department of Regenerative Biomaterials, Nijmegen, The Netherlands; 2 Radboudumc, Department of Plastic, Reconstructive and Hand Surgery, Nijmegen, The Netherlands; 3 Laboratory of Biomaterials and Tissue Engineering, Department Biosciences, Federal University of São Paulo (UNIFESP), Santos, São Paulo Brazil; 4 Department Physiotherapy, Biotechnology Post-Graduate Program, Federal University of São Carlos(UFSCar), São Carlos, São Paulo Brazil

## Abstract

Osteoporosis represents a major health problem in terms of compromising bone strength and increasing the risk of bone fractures. It can be medically treated with bisphosphonates, which act systemically upon oral or venous administration. Further, bone regenerative treatments in osteoporotic conditions present a challenge. Here, we focused on the development of a synthetic bone substitute material with local diminishing effects on osteoporosis. Composites were created using calcium phosphate cement (CPC; 60 wt%) and polylactic-co-glycolic acid (PLGA; 40 wt%), which were loaded with alendronate (ALN). *In vitro* results showed that ALN-loaded CPC/PLGA composites presented clinically suitable properties, including setting times, appropriate compressive strength, and controlled release of ALN, the latter being dependent on composite degradation. Using a rat femoral condyle bone defect model in osteoporotic animals, ALN-loaded CPC/PLGA composites demonstrated stimulatory effects on bone formation both within and outside the defect region.

## Introduction

Osteoporosis is the most frequent human metabolic bone disorder affecting over 75 million people in Europe, Japan and the USA^1^. As such, osteoporosis represents a major health problem in terms of compromising bone strength and increasing the risk of bone fractures^2^. In patients suffering from osteoporosis, the bone healing process is negatively influenced^3^, which challenges the treatment of bone defects or bone fractures. Bone grafting is a widely-used therapy to reconstruct bone defects, although it remains a challenging problem particularly for compromised conditions such as osteoporosis. Autologous bone transplantation can be employed to regenerate bone defects, but its use is hampered by the need for additional surgical procedures and potential limitations in bone quality and quantity. Alternatively, the use of allografts and xenografts is related to immunological issues and an increased risk of cross-species disease transmission^4,5^. In contrast, the use of synthetic biomaterials as a bone substitute has many advantages, including off-the-shelf availability in various shapes and sizes. Still, the biological performance of synthetic bone substitutes is inferior to autologous bone^6^. Consequently, optimization of the biological performance of synthetic bone substitutes via e.g. the release of biologically active factors is a highly explored field within bone biomaterials research^7^.

Osteoporosis is characterized by reduced bone mineral density causing lower bone mass and deterioration of bone tissue micro-architecture. This results from an imbalance between the continuous bone formation and resorption during bone remodeling and an altered variety of proteins in the extracellular matrix of the bone^8,9^. Osteoporosis is primarily observed after menopause in women, with increasing age in both women and men (ratio of 2:1), or secondary by chronic predisposing medical problems^10^. World-wide life expectancy has increased over the last couple of decades for both men and women, resulting in an aging population^1,2,11,12^. This increase in elderly people is expected to continue, ultimately resulting in a further increased need of reliable bone grafting materials for elderly, osteoporotic patients.

The most prescribed medication for osteoporosis are bisphosphonates^13^. The regular route of administration is oral, for which it has been observed that 24 h excretion levels by the renal system reach percentages of 38% to 73%^14^. The major disadvantage of oral administration of bisphosphonates is their poor absorption from the gastro-intestinal tract, generally less than 1%^15^. This is futher negatively influenced when the stomach contains food or has the presence of calcium^15^. The bisphosphonate fraction remaining in the body predominantly localizes to areas with high bone turnover by exploiting its affinity to mineral within the bone extracellular matrix. After localizing to these areas, bisphosphonates can remain within the bone for many years. Among the different types of bisphosphonates, ALN and risedronate are frequently chosen because these both increase bone mineral density, reduce the risk of fracture, and are well tolerated^16^.

In view of the aforementioned, optimization of synthetic bone substitutes with bisphosphonates to locally adjust the imbalance in bone remodeling seems a straightforward approach to aid bone regeneration in osteoporotic conditions. Among the currently available synthetic bone substitutes, calcium phosphate (CaP) based materials are inorganic biomaterials that show high similarity to bone tissue by resembling the mineral phase of bone and are already popular implant materials in several fields of surgery^17^. Calcium phosphate cement (CPC) represents a suitable scaffold material for bone regenerative treatment^18,19^. CPC has the advantage of being injectable and moldable, for which it allows perfect filling directly into a bone defect or by pre-setting it in a specifically designed mold fitted to the defect requirements. When adding porogens, (e.g. poly(lactic-co-glycolic acid) (PLGA) microspheres) to CPC, controlled degradation of the material can be obtained as well as the possibility to release bioactive agents, e.g. osteoinductive growth factors or therapeutic drugs^18,20^. Previous *in vitro* studies proved a successful incorporation of bisphosphonates into ceramic bone substitutes^21–33^. Generally, these studies showed altered but still acceptable material properties, steady release of the bisphosphonate with inhibition of osteoclasts and stimulation of osteoblast. Only a few reported on ceramic implants used *in vivo*^34–37^. These all showed positive results of the material on bone formation.

Here, we used the anti-osteoporotic drug ALN to functionalize CPC/PLGA. We characterized the material morphologically and crystallographically, analyzed setting time and mechanical properties, and determined ALN release kinetics. In addition, we used a femoral condyle bone defect model in osteoporotic rats to comparatively evaluate the biological performance of ALN-loaded CPC/PLGA. We hypothesized that loading CPC/PLGA composites with ALN would (i) not critically affect material morphology and crystallography, (ii) promote controlled ALN release, and hence (iii) enhance the biological performance of CPC/PLGA in osteoporotic conditions by increasing bone formation rate and volumes.

## Results

### Material characterization

**Figure 1:**
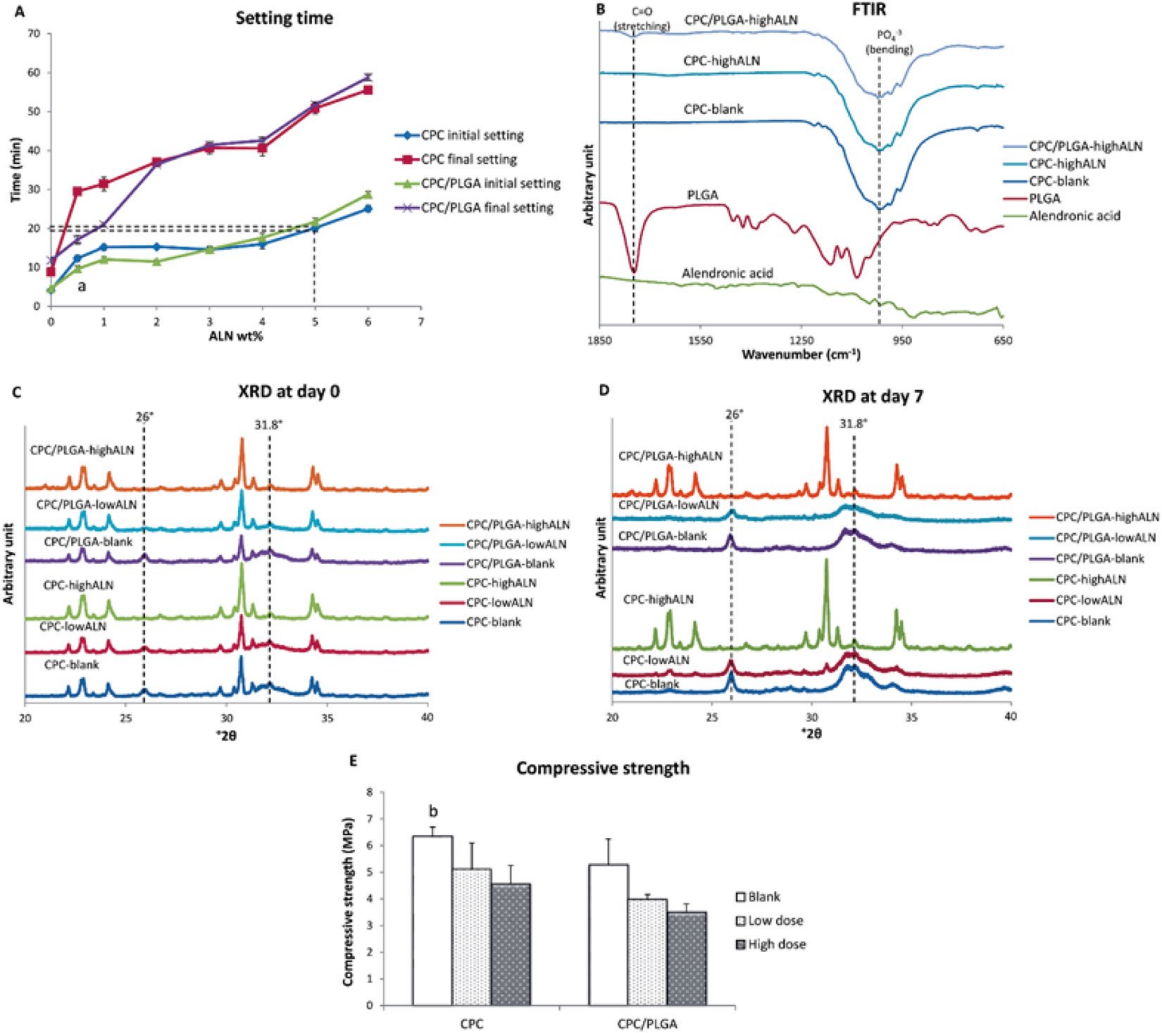
(**A**–**E**) Characterization analysis for CPC/PLGA-ALN composites. (**A**) initial and final setting time at different ALN wt%, dashed lines indicate the initial setting time at 5 wt% ALN for CPC and CPC/PLGA, ^a^p < 0.05 comparing CPC initial setting time with CPC/PLGA initial setting time; (**B**) FTIR of Alendronic acid, PLGA, CPC, CPC-highALN and CPC/PLGA-highALN. Dashed lines indicate peaks associated to PLGA (C=O stretching) and CPC (PO_4_^−3^ bending) in the materials. (**C**,**D**) XRD at days 0 and 7 for the materials, dashed lines indicate HA peaks (26 and 31.8 °2θ); (**E**) compressive strength, ^b^p < 0.05 for CPC-blank compared to CPC/PLGA-highALN. Error bars represent the SD.

After mixing both CPC and CPC/PLGA materials with ALN, all formulations remained injectable using standard syringes. Setting times (Fig. 1A) of CPC and CPC/PLGA formulations showed to range from 4 to 28 min for initial and from 8 to 58 min for final setting. ALN loading via the liquid phase significantly increased setting times of both CPC and CPC/PLGA formulations. For subsequent *in vitro* experiments, we chose ALN doses of 0.5 wt% (low dose) and 5.0 wt% (high dose), which respectively presented initial setting times of ~10–12 min and ~20–22 min for both CPC formulations.

**Figure 2:**
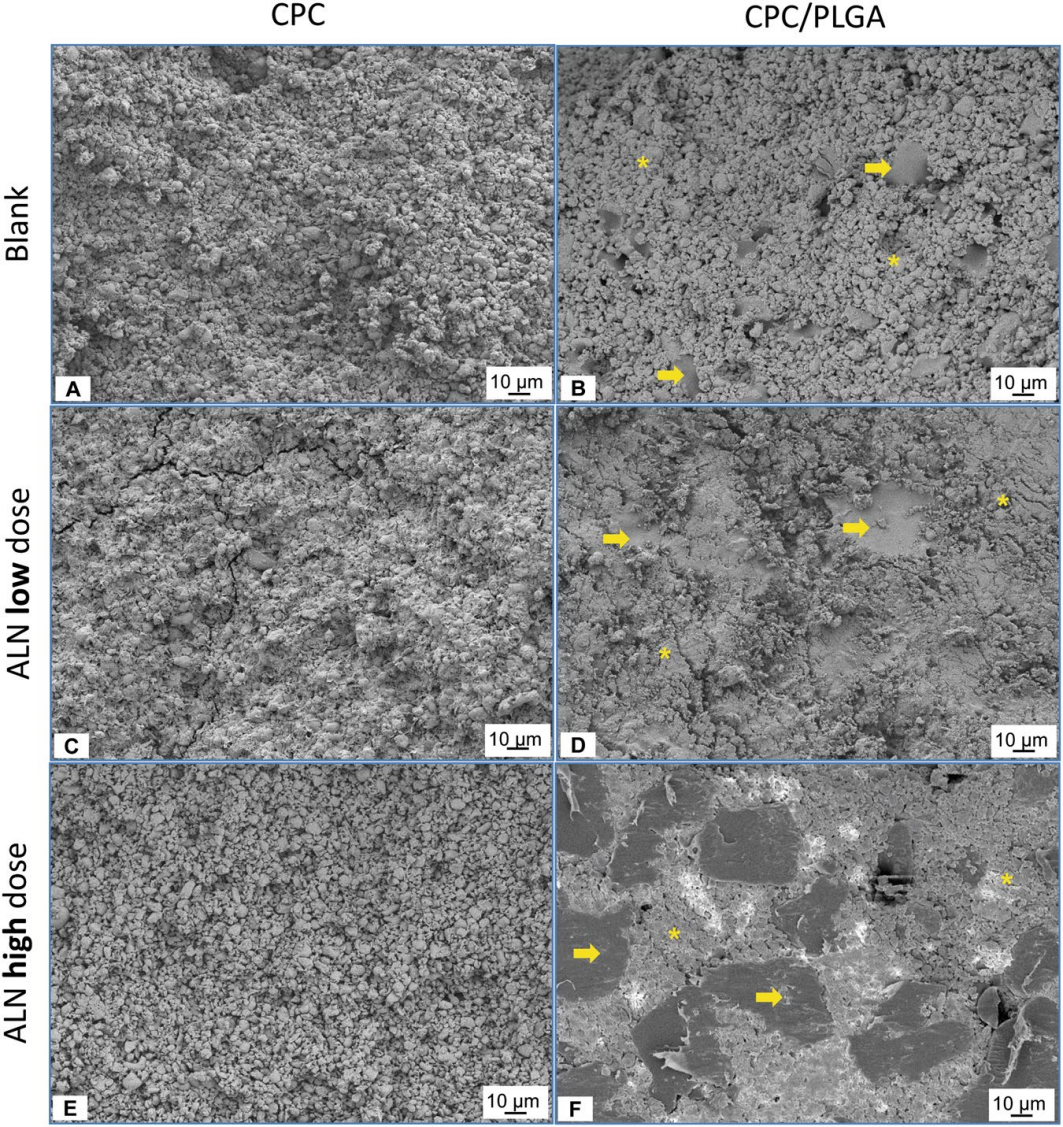
(**A–F**) Micrographs obtained by SEM. (**A**) CPC-blank. (**B**) CPC/PLGA-blank. (**C**) CPC-lowALN. (**D**) CPC/PLGA-lowALN. (**E**) CPC-highALN and (**F**) CPC/PLGA-highALN. Asterisks and arrows indicate CPC and PLGA particles respectively. Magnification of 500×.

Figure 2 shows SEM micrographs for all CPC formulations. PLGA could be discriminated due to morphological and size differences compared to the CPC matrix. The composites presented a homogenous aspect with PLGA particles dispersed throughout the CPC matrix (Fig. 2B,D and F).

FTIR analysis (Fig. 1B) showed absorption peaks representative for α-TCP and PLGA. A strong peak of carbonyl at ~1750 cm^−1^ was detected in PLGA containing samples, while representative peaks for PO_4_^−3^ at the range of 900 to 1100 cm^−1^ were observed for all formulations. Due to low crystallinity of ALN, no characteristic peaks were observed for this component.

XRD patterns of the composites at day 0 (after setting) showed an initial phase transition from α-TCP to hydroxyapatite (HA; Fig. 1C; dashed lines) for CPC-blank, CPC-lowALN, CPC/PLGA-blank and α-CPC/PLGA-lowALN. After 3 and 7 days of incubation, peaks for HA formation were observed (at ~26 and 31.8 °2θ; Fig. 1D), but no HA peaks were observed for CPC-highALN and CPC/PLGA-highALN, indicating that phase transformation was not taking place for high ALN formulations. This pattern was also observed after 148 days (data not shown).

The compressive strength for CPC and CPC/PLGA formulations assessed by mechanical test ranged from ~4 to 6 MPa (Fig. 1E). The addition of ALN to CPC or CPC/PLGA tended to decrease the compressive strength for CPC and CPC/PLGA formulations. Statistical differences were found for CPC-blank compared to CPC/PLGA-highALN (p = 0.0115).

### ALN release kinetics

**Figure 3:**
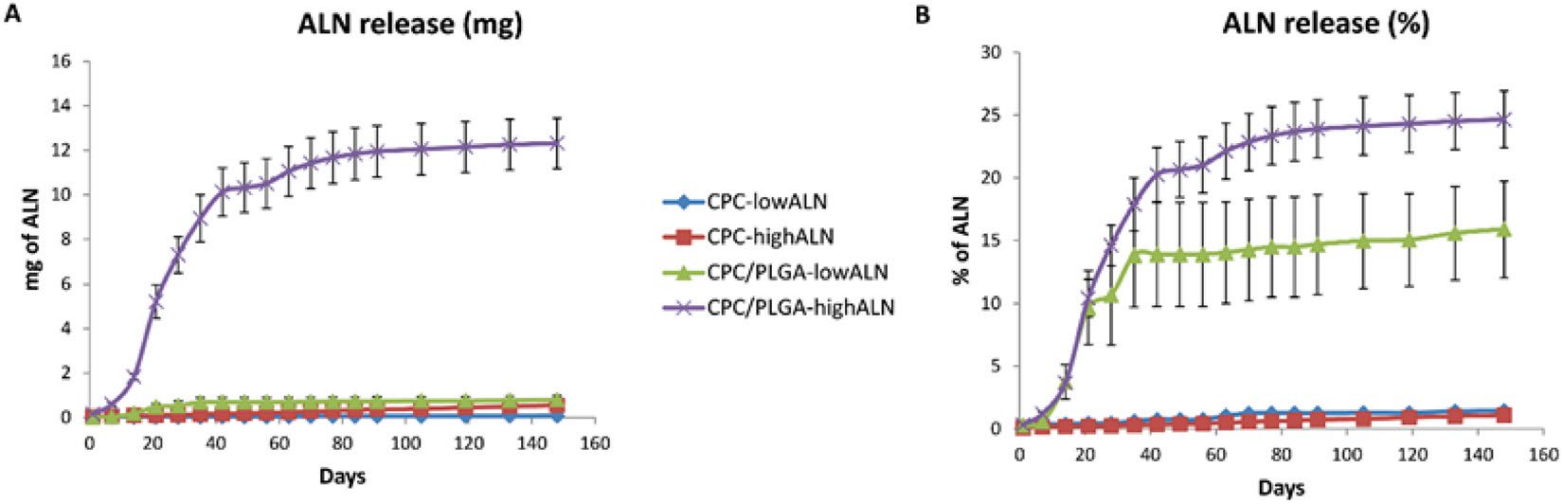
(**A,B**) Cumulative alendronate release from CPC and CPC/PLGA cylinders loaded with the bisphosphonate for up to 148 days. (**A**) Absolute ALN release (mg). (**B**) Relative ALN release (% of loaded dose). Error bars represent the SD.

The ALN release data assessed via ninhydrin assay showed negligible ALN release for CPC-formulations (Fig. 3). In contrast, the CPC/PLGA formulations showed a 2-week lag phase with negligible release followed by a biphasic ALN release with 2 distinct sustained release phases. A first fast and sustained ALN release from day 15 to 42 (~0.02 and ~0.20 mg ALN/day for low and highALN, respectively) reached ~15% (~0.7 mg) and 20% (~10 mg) of the initially loaded ALN amount for CPC/PLGA-lowALN and CPC/PLGA-highALN, respectively. Thereafter, a second sustained release phase with lower ALN release was observed (~9.40 × 10^−4^ and ~0.02 mg ALN/day for low and highALN, respectively), which after 148 days cumulatively reached ~16% (~0.8 mg) and 25% (~12.0 mg) of the initially loaded ALN amount for CPC/PLGA low and high dosages, respectively.

Based on the *in vitro* data of ALN incorporation into CPC and CPC/PLGA, we proceeded with *in vivo* analysis using only degradable CPC/PLGA, since no ALN release was detected from CPC. Additionally, CPC/PLGA-highALN was excluded for *in vivo* studies, as it showed incomplete phase transformation (based on setting time and physicochemical analysis).

### Biological performance

#### Micro-CT analysis

**Figure 4:**
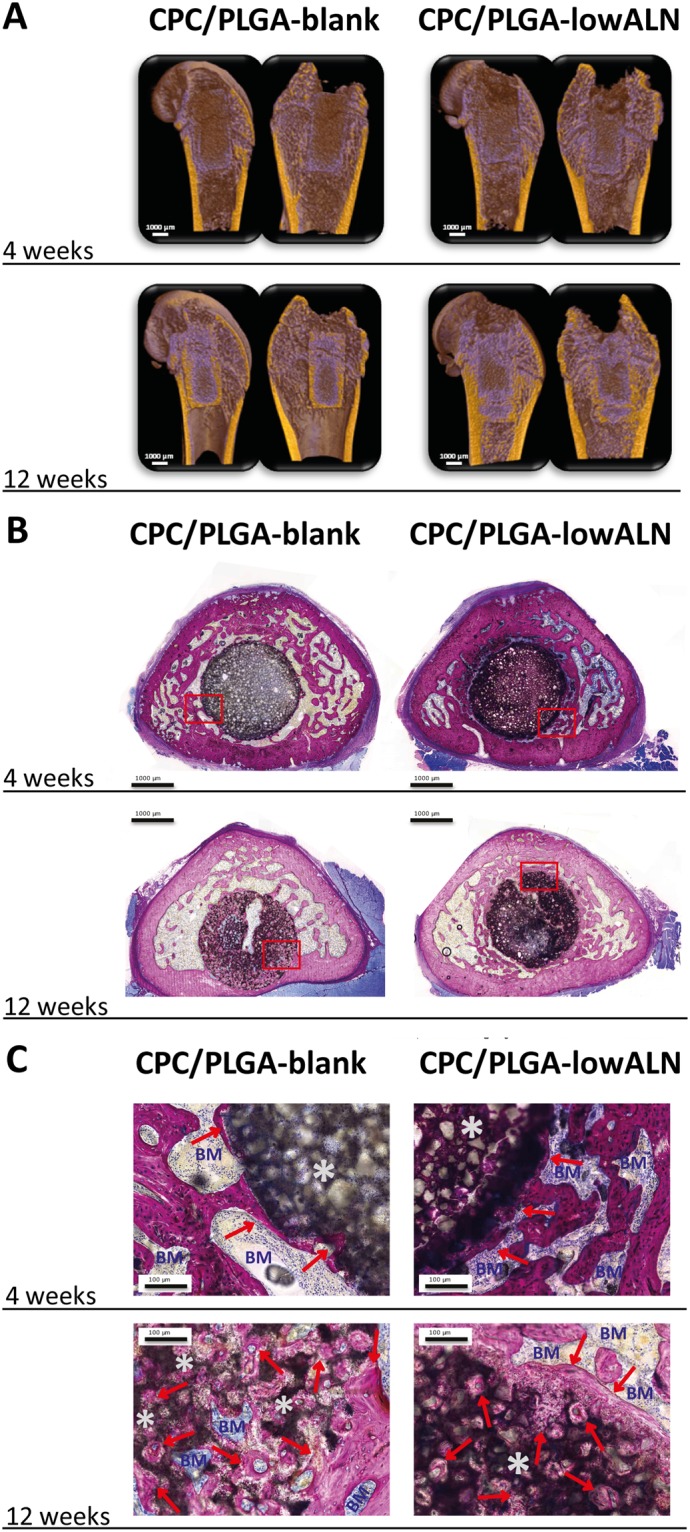
(**A–C**): (**A**) Representative micro-CT images of each experimental group. Color settings were adjusted to distinguish between different densities of bone and material, coloring bone yellow and CPC/PLGA purple. Representative histological images of pMMA sections for each experimental group with images at (**B**) 4 weeks and 12 weeks and (**C**) corresponding images at high magnification; methylene blue and basic fuchsine staining; red arrows] new bone (pink color); *CPC/PLGA; BM] bone marrow (blue color).

The micro-CT data were reconstructed to 3D representations and then sliced to obtain an internal view of the bone defects. A representative overview with sagittal, frontal and transversal slices through the defect region for each experimental group is presented in Fig. 4A. Color settings were adjusted to distinguish between different densities of bone and material. After 4 and 12 weeks, both CPC/PLGA-blank and CPC/PLGA-lowALN showed presence of CPC in contact with surrounding bone tissue. With implantation time, both CPC/PLGA-blank and CPC/PLGA-lowALN showed more bone formation around the composites.

#### Descriptive histology

Representative images of the histological sections of specimens embedded in pMMA are depicted in Fig. 4B. In general, histological analysis showed differential material degradation and bone formation depending on the composition of the implant. For CPC/PLGA-blank and CPC/PLGA-lowALN, no apparent degradation was visible at 4 weeks, and peripheral degradation was observed after 12 weeks. There was an increased amount of bone formation, macroscopically, around the composites for CPC/PLGA-lowALN compared to CPC/PLGA-blank. Similar as with the micro-CT images, this difference was more apparent after 12 weeks. The histological slides of CPC/PLGA-lowALN further showed an apparently higher bone density outside the ROI. At higher magnification (Fig. 4C), newly formed bone in direct contact with CPC/PLGA-blank and CPC/PLGA-lowALN was observed. Within the ROI, both CPC/PLGA-blank and CPC/PLGA-low ALN showed that bone had reached the center of the ROI.

#### Bone dynamics by fluorochrome labeling

**Figure 5:**
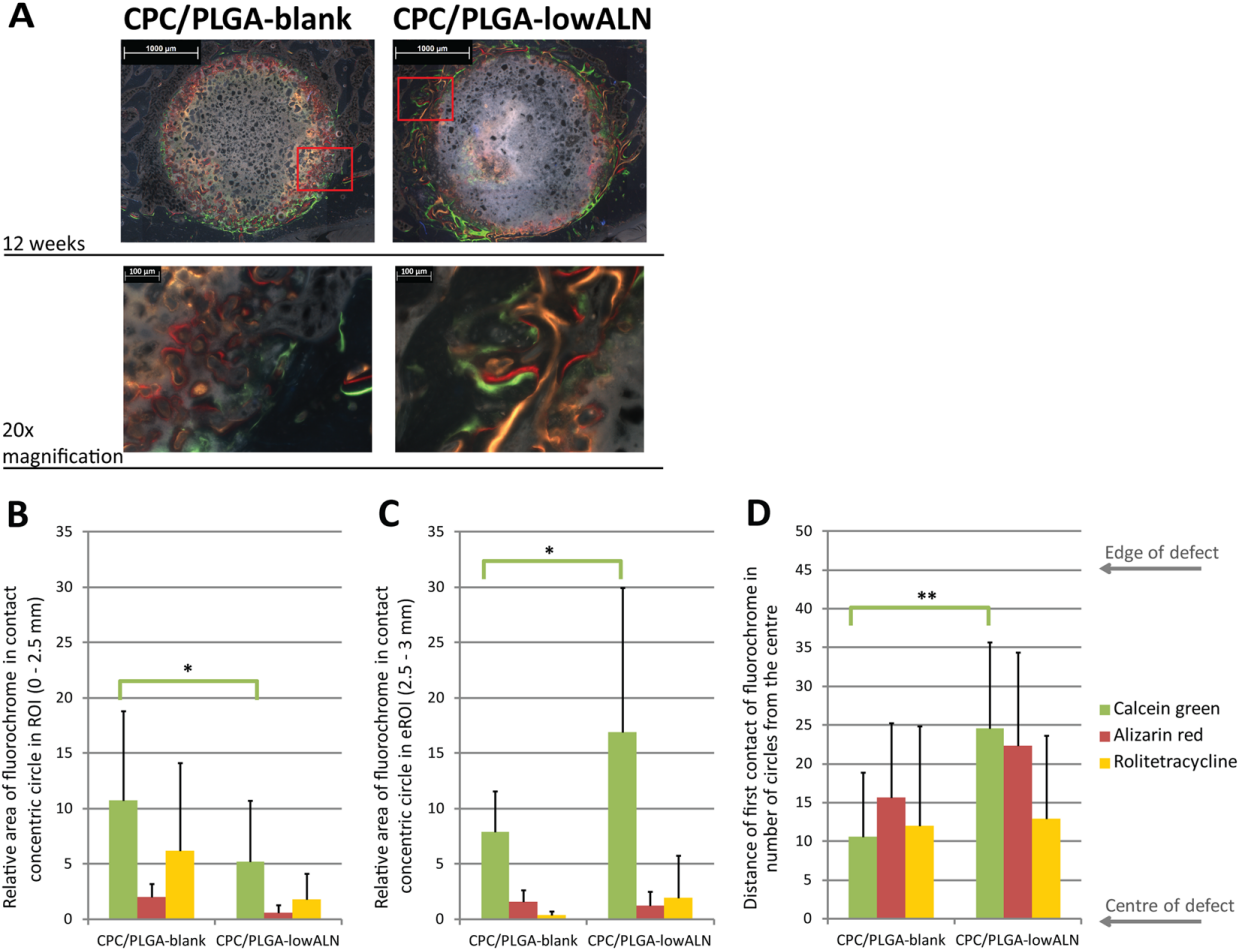
(**A–D**): (**A**) Representative fluorochrome microscopy images at 12 weeks, with high magnification images; red box] area of high magnification; green color] calcein green (week 4); red color] alizarine complexone (week 6); yellow color] rolitetracycline (week 8). (**B–D**): Relative volume areas (mean + *SD*) of fluorochrome labels in contact with concentric circles for calcein green (week 4), alizarine complexone (week 6) and rolitetracycline (week 8) for both CPC/PLGA-blank and CPC/PLGA-lowALN (**B**) within ROI (0–2.5 mm) and (**C**) extended ROI (eROI, 2.5–3 mm); statistical analysis showed significantly more calcein green within ROI for CPC/PLGA-blank compared to CPC/PLGA-lowALN (Student’s t-test, p < 0.05); analysis showed significantly more calcein green in the eROI for CPC/PLGA-lowALN compared to CPC/PLGA-blank (Student’s t-test, p < 0.05); (**D**) Distance of the first ring that is in contact with a fluorochrome (mean + *SD*) for both CPC/PLGA-blank and CPC/PLGA-lowALN; statistical analysis showed significantly closer contact with calcein green for CPC/PLGA-blank compared to CPC/PLGA-lowALN (Student’s t-test, p < 0.01); Range] 0 to 55 rings, edge of defect] ring 45 (2.5 mm from centre).

Incorporation of fluorochrome labels into newly formed bone (Fig. 5A) was observed for calcein green (green; week 4), alizarine complexone (red; week 6) and rolitetracycline (yellow; week 8). However, none of the sections showed incorporation of calcein blue (blue; week 2). For both CPC/PLGA-blank and CPC/PLGA-lowALN, calcein green was mostly present within the ROI (Fig. 5B). This was significantly more for CPC/PLGA-blank compared to CPC/PLGA-lowALN (p < 0.05). However, in the peripheral area of 2.5 to 3 mm from the center of the ROI (Fig. 5C), CPC/PLGA-lowALN showed significantly higher presence of calcein green compared to CPC/PLGA-blank (p < 0.05). Bone formation dynamics by fluorescent microscopy showed that significantly more bone was formed near the center of the defect at 12 weeks for CPC/PLGA-lowALN compared to CPC/PLGA-blank (p < 0.01; Fig. 5D).

#### Histomorphometrical analysis

**Figure 6:**
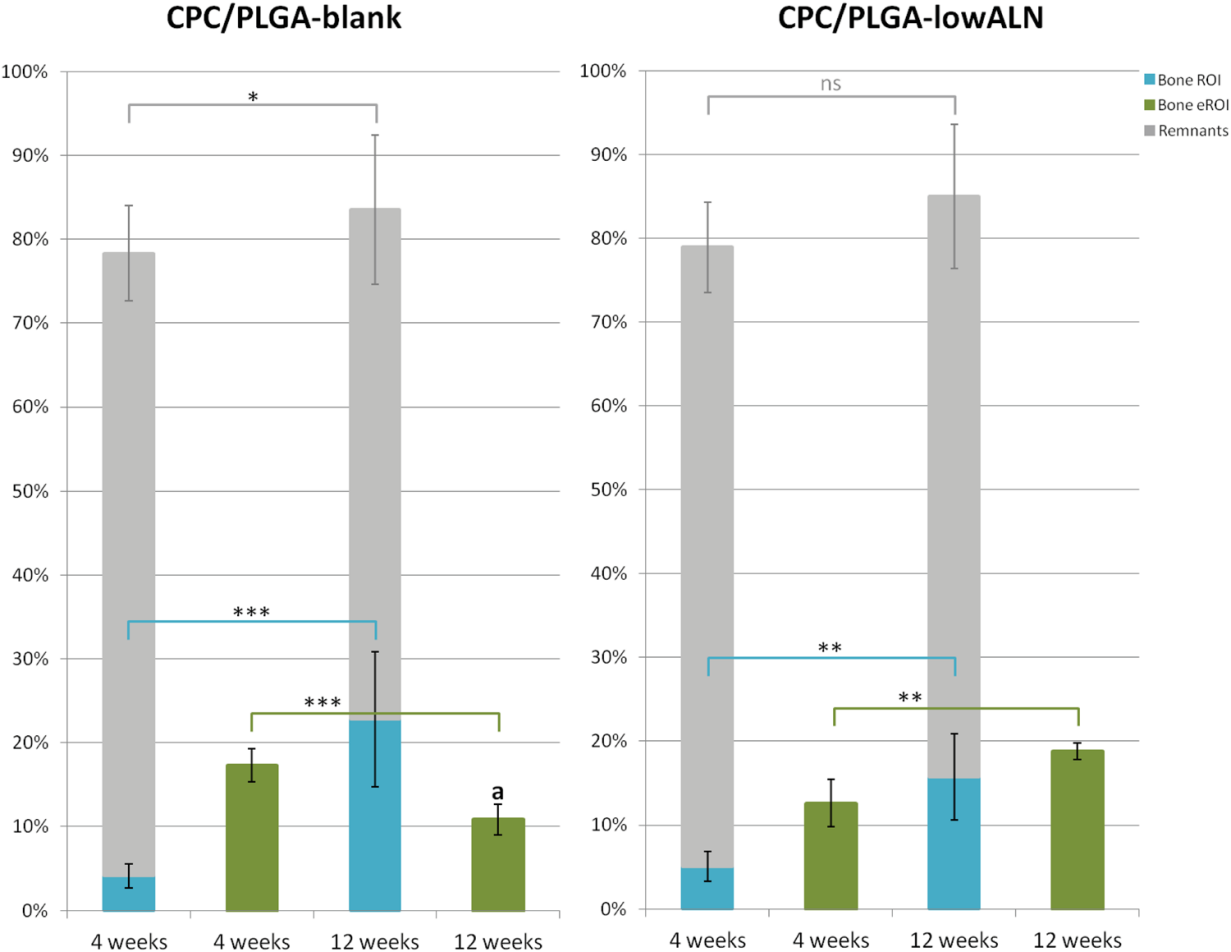
Accumulated volume percentages (mean ± *SD*) of new bone formation (green bars for eROI and blue bars within ROI) and material remnants (grey bars) at 4 and 12 weeks after implantation for CPC/PLGA-blank and CPC/PLGA-lowALN; statistical analysis showed significant increase of bone formation within ROI from 4 to 12 weeks for both CPC/PLGA-blank and CPC/PLGA-lowALN (***p < 0.001, **p < 0.01); bone formation over time in the eROI significantly decreased for CPC/PLGA-blank (p < 0.001), but significantly increased for CPC/PLGA-lowALN (p < 0.001); material remnants significantly decreased for CPC/PLGA-blank (*p < 0.05) but not significantly for CPC/PLGA-lowALN (ns = p > 0.05); ^a^At 12 weeks there was a significant higher amount of bone in the eROI for CPC/PLGA-lowALN compared to CPC/PLGA-blank (p < 0.05).

Histomorphometry data (Fig. 6) showed CPC remnants within the ROI after 4 weeks of 74.2 ± *5.7*% for CPC/PLGA-blank and 71.7 ± *5.4*% for CPC/PLGA-lowALN. After 12 weeks, CPC remnants within the ROI were 60.7 ± *8.9*% for CPC/PLGA-blank and 66.5 ± *7.1*% for CPC/PLGA-lowALN. Statistical analysis showed significant temporal decrease from 4 to 12 weeks of material remnants was seen for CPC/PLGA-blank (p < 0.05), but not for CPC/PLGA-lowALN (p > 0.05).

**Table 1:** Labels and descriptions of the experimental groups.

Labels	Descriptions
CPC-blank	Only α-TCP
CPC-lowALN	α-TCP + 0.5 wt % ALN
CPC-highALN	α-TCP + 5.0 wt % ALN
CPC/PLGA-blank	60% α-TCP/40% PLGA
CPC/PLGA-lowALN	60% α-TCP/40% PLGA + 0.5 wt % ALN
CPC/PLGA-highALN	60% α-TCP/40% PLGA + 5.0 wt % ALN

Bone areas within the ROI after 4 weeks were 4.1 ± *1.4*% for CPC/PLGA-blank and 5.5 ± *1.4*% for CPC/PLGA-lowALN. After 12 weeks, bone areas within the ROI were 22.8 ± *8.0*% for CPC/PLGA-blank and 16.4 ± *4.1*% for CPC/PLGA-lowALN. Statistical analysis for bone area within ROI showed no significant difference between CPC/PLGA-blank and CPC/PLGA-lowALN for both time points (p > 0.05). Data showed significant temporal increase from 4 to 12 weeks of bone for CPC/PLGA-blank and CPC/PLGA-lowALN (Student’s t-test, both p < 0.001).

Descriptive histology suggested increased bone formation outside the ROI, therefore, bone areas were also measured in an extended ROI (eROI, from 2.5 to 3 mm). After 4 weeks bone area outside the ROI were 17.3 ± *2.0*% for CPC/PLGA-blank and 15.5 ± *3.7*% for CPC/PLGA-lowALN. Bone area for the eROI after 12 weeks, were 10.9 ± *1.8*% for CPC/PLGA-blank and 18.1 ± *2.1*% for CPC/PLGA-lowALN. Statistical analysis for bone area for the eROI showed significantly more bone at 12 weeks there was significantly more bone for CPC/PLGA-lowALN compared to CPC/PLGA-blank (Student’s t-test, p < 0.05). Data showed significant temporal increase from 4 to 12 weeks of bone only for CPC/PLGA-lowALN (Student’s t-test, p < 0.01). There was significantly less bone formation outside the ROI for CPC/PLGA-blank at 12 weeks compared to at 4 weeks (p < 0.001).

## Discussion

The aim of this study was to develop CPC/PLGA composites loaded with ALN and investigate the morphological features, physicochemical properties and ALN release kinetics. Furthermore, we evaluated the efficacy of CPC and CPC/PLGA as a local ALN release system in a rat femoral condyle bone defect in osteoporotic rats. We hypothesized that addition of ALN would (i) not critically affect material physicochemical properties, (ii) promote controlled ALN release, and (iii) enhance the biological performance of the CPC for bone regeneration in osteoporotic rats. We showed that ALN loading increased CPC and CPC/PLGA setting times and marginally affected compressive strength. Loading ALN at a dose of 5 wt% impeded with the conversion of the alpha-TCP base powder to HA within 3 days of incubation in PBS, while a dose of 0.5 wt% did not. ALN release kinetics showed hardly any release from CPC, whereas CPC/PLGA showed a controlled release of ALN up to 148 days with 2 consecutive sustained release phases following a 2-week lag phase. The main *in vivo* findings by micro-CT imaging, descriptive histology and quantitative histomorphometry showed that loading of ALN within CPC/PLGA did not increase bone formation within the defect region, but enhanced bone formation within the peri-defect region compared to control CPC/PLGA.

Previous *in vitro* studies proved a successful incorporation of bisphosphonates into ceramic bone substitutes^21–32^. These studies showed decrease of the mechanical properties and increases the setting time of the ceramic, however the range was still within acceptable limits^21,26,31,32^. This is consistent with our study and dependent of dose of the bisphosphonate added. By *in vitro* analysis the previous studies showed the release of the drug to be generally steady and not cytotoxic^21,22,27–29,31,32^. Moreover, there was a inhibition of osteoclasts and stimulation of osteoblasts^22–27,29,30^. Most of these studies also used ALN, however there were different ways of incorporation the drug into the cement. Most of the studies used the technique of loading the drug onto the surface of an inorganic implant by soaking the implant in a solution containing the drug^21–25,28–32^. However, this technique has the disadvantage of limited drug loading content and results in a uncontrolled burst release on administration^38^. The study by Panzavolta *et al*. incorporated the drug (both ALN and pamidronate) into the liquid phase, they do not report on the release properties^26^. In the study by Shi *et al*. the ALN was incorporated into the liquid phase of PLGA which was then incorporated as a porogen into the cement^27^. Their results showed a controlled release which is in concurrence with our study. There are previous *in vivo* studies using a cement enhanced with bisphophonate, showing positive results on bone formation^34–37^. However, a fair comparison between these four studies is compromised, due to use of all different cements in combination with other bisphosphonates and also the animal models were different.

For clinical application, handling properties of injectable bone substitutes that harden *in situ* are of utmost importance. Alterations in the composition of CPC or CPC/PLGA have shown to affect important handling properties, including injectability^39^ and setting time^40,41^. Here, addition of ALN into the liquid phase of the cement formulation make it possible to obtain injectable materials that were extruded by standard syringes^42^, which corroborates previous work^43^. For setting time evaluation, we used systematic increases of ALN loading (0.5–6 wt%) and observed clinically acceptable initial setting times (i.e. <20 min)^19^ for ALN amounts up to 5 wt%. ALN act on the CPC hydration process which is a reaction of dissolution-precipitation of CPC particles and Ca^2+^/PO_4_^−3^ ions respectively, affecting the curing of CPC^43^. In view of eventual clinical application, CPC/PLGA composites with low (0.5 wt%) and high (5.0 wt%) doses of ALN were subsequently used for further experimental *in vitro* work. As a less important characteristic for injectable bone substitutes meant for application under minimal loading, ALN loading showed marginal lowering effects on compressive strength. Panzavolta *et al*.^44^ and Li *et al*.^45^ also demonstrated a lowering effect of ALN on the CPC compressive strength, but with values of ~5.0 MPa these remain close to those of human cancellous bone^45^.

From a material perspective, FTIR analysis and XRD confirmed the conversion of the alpha-TCP base powder to HA formed through hydration (HA, JCPDS 01-074-0566)^46^ within 3 days of incubation in PBS for CPC and CPC/PLGA formulations without or low ALN loading. In contrast, high ALN loading inhibited this conversion. It is well-known that α-TCP hydrolyses to hydroxyapatite through dissolution and successive precipitation of the more stable phase, and the high affinity of ALN for calcium ions retards this process^43^.

ALN release kinetics showed negligible release from CPC, whereas CPC/PLGA showed a controlled release of ALN with 2 distinct consecutive sustained release phases after a 2-week lag phase. Apparently, the dense configuration of the CPC and related lack of degradation is responsible for the observed negligible ALN release. Similarly, the relatively dense CPC/PLGA composite configuration upon setting limits ALN release during a 2-week lag phase. Subsequently, sustained ALN release coincides with the generation of a porous network within the ceramic matrix due to PLGA degradation. The second sustained ALN release phase takes place after PLGA degradation from ~day 40 onwards, when liquid can penetrate the ceramic matrix via an interconnective porosity system^47^. The difference in average daily ALN relative release (~20 and ~10 times greater in the 1^st^ compared to 2^nd^ phase for CPC-lowALN and CPC-highALN, respectively) is likely due to the additional acidifying effect of PLGA degradation products in the 1^st^ phase, while a sole dissolution-controlled release process from a porous ceramic matrix remains thereafter^48,49^. Our observations are in accordance with those of Panyam and coworkers^50^, who studied the use of PLGA micro- and nanoparticles for drug-delivery, and previous work from our group using CPC/PLGA as a drug-delivery vehicle^51,52^.

Over the last decade, several studies examined the effect of bisphosphonates on peri-implant bone response using metallic bone implants^53–66^ and showed enhanced bone formation (using mainly ALN and zolendronate) compared to controls without bisphosphonate, with evaluation periods up to one year^59^. This is in accordance with our results, which showed enhanced peri-defect bone formation in response to locally released ALN from CPC/PLGA composites. However, these studies differ in the use of non-degradable metallic implants versus degradable CPC/PLGA in our study. Remarkably, we did not observe any influence of ALN loading on bone formation within the defect region, for which the reason remains unclear.

CPC has the advantage as a bone substitute material of being injectable and moldable, allowing to directly adapt or being pre-set to the shape of a bone defect^18,19^. For this study we used pre-set composite of CPC/PLGA with and without ALN, specifically shaped to the measurements of our defect. With these pre-set CPC/PLGA composites we aimed to ensure the implants contained consistent amounts of cement and alendronate for both our *in vitro* and *in vivo* studies, as well as reduce operative time.

## Conclusion

This study showed by *in vitro* analysis that ALN-loaded CPC/PLGA presents clinically acceptable handling, suitable compressive strength, and a controlled ALN release. The *in vivo* bone defect model showed that a relative low dose of ALN (0.5 wt%) signifcantly increases bone formation in the peri-defect region in osteoporotic conditions, without effects on CPC/PLGA degradation. The data suggest that future clinical application of ALN-loaded CPC/PLGA could be benificial for bone healing in compromised conditions.

## Materials and Methods

### Materials

Alpha tri-calcium phosphate (α-TCP) was provided by Cam Bioceramics B.V. (mean particle ~4.0 µm; Leiden, The Netherlands). Poly(DL-lactic-co-glycolic acid) PLGA particles (containing both a lactic and glycolic weight percentage of 50%; particle size ~60 µm) was obtained from Corbion (PLGA, Purasorb^®^, Gorinchem, The Netherlands). Carboxymethylcellulose (CMC) was purchased from Kelco (Georgia, USA) and alendronate (ALN) was purchased from AK Scientific (Union City, California, USA).

#### Preparation of CPC/PLGA and CPC/PLGA-ALN composites

CPC/PLGA composites were prepared by weighing 0.597 g of α-TCP, 0.398 g of PLGA and 0.005 g of CMC in a beaker. Subsequently, the liquid component (4% NaH_2_PO_4_ solution) was added in a liquid/power ratio of 0.50 and the composition was mixed with a spatula. Afterwards, the composite was injected, using a 2 ml syringe (BD Plastipakt, Becton Dickinson S.A., Madrid, Spain), into teflon molds to obtain cylinders of 5 mm × 3 mm (diameter and height) for *in vitro* analysis. For mechanical tests, cylinders of 6 mm × 12 mm (diameter and height) were prepared utilizing different molds.

ALN was introduced into CPC/PLGA scaffolds via the liquid CPC component. Briefly, ALN was dissolved in 4% NaH_2_PO_4_ at different concentrations (range: 0–6 wt% for setting time investigations) and the pH was adjusted to ~7.4. The selected experimental groups are presented in Table 1.

For *in vivo* experiments, pre-set cylindrical CPC/PLGA composites (2.5 mm in diameter and 5 mm in height) were prepared, either or not containing ALN (2 μg/ml for the CPC/PLGA-lowALN) within the liquid component. This concentration corresponds to 0.5 wt.% ALN. After setting for 24 h at room temperature, composites were removed from the mold and sterilized using ethylene oxide (Synergy Health, Venlo, The Netherlands).

### Material characterization

#### Setting time

The setting time of CPC-formulations was determined using the Gillmore needle method at 37 °C, as described previously (ASTM C266, 1999)^67^. For this purpose, a bronze block containing six holes was used as a mold (6 mm in diameter, 12 mm in height). The different formulations (n = 3) were inserted into the mold in order to assess the initial and final setting.

#### Morphology

Immediately after setting, the samples were mounted on stubs with carbon tape and sputter coated with gold. SEM (SEM, JEOL 6310) was performed to examine the structural morphology of the composites.

#### Physico-chemical analysis

Immediately after preparation and setting, the samples were pulverized and submitted to Fourier transform infrared spectroscopy (FTIR; Perkin-Elmer 1700, UK). FTIR was performed to characterize the chemical bonds present in the samples. Analyses were done using pulverized samples in the range of 520–4000 cm^−1^ with a resolution of 2 cm^−1^. The samples were scanned 100 times for each FTIR measurement and the spectrum acquired was the average of all these scans.

X-ray diffraction (XRD, Cu-Ka, 45 kV, 30 mA, Philips BV, the Netherlands) patterns of pulverized samples were collected (2θ range: 20–40°) to crystallographically characterize the phases. XRD evaluation was done at days 0, 3, 7 and 148 after incubation in 1.0 ml of phosphate buffered saline solution (PBS; pH 7.4).

#### Mechanical testing

After setting, the compressive strength (CS) of the CPC cylinders was measured, in the longitudinal direction of the specimens, at a loading rate of 1 mm/min using a testing bench machine (Model 858, Mini-Bionix II, MTS Systems Corp., Eden Prairie, MN, USA) using three cylinders per experimental group (n = 3).

#### ALN release from CPC/PLGA-ALN composites

*In vitro* release tests were performed by soaking one sample cylinder of each CPC formulation in 1.0 ml of PBS and incubating the samples (n = 3) under agitating conditions at 37 °C. ALN release was determined using a ninhydrin assay on releasates collected after selected time periods (range: 1–148 days)^68^. Briefly, 100 µL of releasate (complete removal of 1 ml and addition of 1 ml of fresh PBS) or standard (serial dilutions of ALN; range: 0–200 µM) was pipetted in a 96-well plate, after which 75 µL of ninhydrin color reagent (Sigma-Aldrich, St. Louis, USA) was added for incubation at 80 °C for 30 min. Subsequently, the well plate was cooled and 100 µL of stabilizing solvent (50% ethanol) was added into each well. The absorbance of each well was measured on a microplate spectrophotometer at 570 nm (Bio-Tech Instruments, Winooski, VT, USA).

### Biological performance

#### Animals

For this experiment, twelve healthy mature female Wistar rats were used at 3 months of age with an average weight of ~200 g. Animals were allowed to acclimatize for 7 days and housing was provided per pair in a standard macrolon type 3 cage. To induce an osteoporotic condition, rats were subjected to bilateral ovariectomy (OVX). All rats received two surgical procedures during the course of the experiment: OVX surgery and 6 weeks later bone defect surgery. After OVX surgery, a low calcium diet was provided during six weeks with pellets containing 0.01% calcium and 0.77% phosphorous (Ssniff Spezialdiäten GmbH, Soest, Germany). After the bone defect surgery, a standard rodent chow was provided ad libitum. The housing room was maintained under standard laboratory conditions (light-dark cycle: 12:12 h, temperature: 20–22 °C, relative humidity: 45–55%). All experiments were conducted in accordance with institutional, national and international guidelines for animal care and the Dutch law concerning animal welfare. The studies were reviewed and approved by the Experimental Animal Committee of the Radboud University (RUDEC 2013-080).

#### Surgical procedure to induce osteoporotic conditions

All animals received OVX surgery through two separate incisions in the lateral abdominal wall. The same procedure was followed as previously described by Alghamdi *et al*.^69^.

#### Surgical procedure to create and fill femoral bone defects

Six weeks after OVX surgery, a bone defect was created in both femoral condyles of each animal. Alternating the left and right femur, CPC/PLGA-blank and CPC/PLGA-lowALN, pre-set composites were used to fill the defects. Anesthesia was induced and maintained by Isoflurane inhalation (Rhodia Organique Fine Limited) combined with oxygen delivered by mask. Pre-operatively pain medication was provided by an injection of Carprofen (Rimadyl®, Pfizer Animal Health, New York, USA) in dosage of 5 mg/kg, given 15 min before surgery. For a minimum duration of 2 days postoperatively, Carprofen was given every 24 h. Additionally, to diminish postoperative pain, Buprenorfine (Temgesic®, Reckitt Benckiser Health Care Limited, Schering-Plough, UK) was given every 12 h in a dosage of 0.01 mg/kg. Additionally, to ensure adequate postoperative analgesia, before closing the wound local drop anaesthesia was provided by Lidocaïne (10 mg/ml Lidocaïne FNA, Centrafarm B.V., Etten-Leur, the Netherlands) and Bupivacaïne (5 mg/ml Bupivacaïne Actavis, Actavis B.V., Baarn, the Netherlands) diluted with NaCl using ~1 ml per rat containing 1 mg of Lidocaïne and 0.25 mg of Bupivacaïne.

Before creating femoral defects, both hind limbs of the rats were shaved and disinfected with povidone iodine. The rats were immobilized in supine position and with the knee maximally flexed a longitudinal incision through skin and muscle was made on the medial surface. After exposure of the medial side of the distal femoral condyle, the knee capsule was incised. Then, the knee was extended to luxate the patella laterally. When a clear view of the knee joint was established, a bone defect (Ø 2.5 mm, depth 5 mm) was created longitudinal to the axis of the femur, using a dental drill (Elcomed 9927 SPS; W&H Dentalwerk Burmoos GmbH, Burmoos, Austria). A series of increasing bur diameters (1.5–2.0–2.3–2.5 mm) were used at a speed of maximum 5000 rpm and a constant cooling by dripping saline. The drilled cavity was then washed with saline and dried using sterile gauze.

Subsequently, the CPC/PLGA composites were alternatingly placed into the defect. A total of 24 implants were used, resulting in n = 6 per group. To increase statistical power paired analysis was made possible by combining CPC/PLGA-blank with CPC/PLGA-lowALN in each rat. After local drop anesthesia, the muscular tissue layer was closed with absorbable sutures (Vicryl® 4.0, Ethicon, Amersfoort, The Netherlands) after which the skin was closed by small staples (Agraven®; InstruVet BV, Cuijk, The Netherlands). In the initial postoperative period, the intake of water and food was monitored as well as the weight of the animals. In addition, the animals were observed for signs of pain, infection and proper activity and weighed again postoperatively once a week to identify significant weight loss (>20%) compared to preoperative body weight of each rat.

#### Fluorochrome labeling for bone dynamics analysis

Animals assigned to a 12-week implantation period received a series of four fluorochromes (SigmaAldrich; Munich, Germany) by subcutaneous injection with a 2-week interval, starting two weeks after implantation surgery: Calcein blue (product no. M1255, dose 30 mg/kg; week 2), calcein green (C0875, 10 mg/kg; week 4), alizarine complexone red (A3882, 25 mg/kg; week 6) and rolitetracycline yellow (R2253, 25 mg/kg; week 8). Before injection, the solutions were set to neutral pH (7.2–7.4), then filtered through a 0.22 sterile millipore filter, and finally checked for fluorescence.

#### Specimen retrieval

After implantation periods of 4 and 12 weeks, the animals were euthanized by suffocation with CO_2_. The femurs including the implanted materials were harvested, stripped from soft tissues and immediately fixed in 10% formalin solution. Using a diamond saw, the specimens were cut to become suitable for *ex vivo* micro-CT scanning and histological processing. After fixation, specimens were stored in 70% ethanol.

#### Micro-CT

A desktop X-ray micro-CT system was used (Skyscan-1072, TomoNT version 3 N.5, Skyscan®, Kontich, Belgium) for 3D imaging. The specimens were placed onto the sample holder with the long axis of the femur perpendicular to the scanning beam. 3D reconstruction and imaging processing was performed using NRecon V1.4 and CTvox v2.7 (Skyscan®, Kontich, Belgium), respectively.

#### Histological processing

After performing micro-CT scanning, the specimens were dehydrated in a graded series of ethanol (70%-100%), after which they were embedded in polymethylmethacrylate (pMMA). After polymerization, thin sections of 10 μm were prepared in a cross-sectional direction perpendicular on the longitudinal direction of the defect using a diamond blade microtome (Leica Microsystems SP 1600, Nussloch, Germany) using a sawing technique as previously described^69,70^. Using this microtome sawing technique, at least three sections of ~10 μm thick per specimen are cut with intermediate loss of ~300 μm (thickness of sawing blade). With this loss the sections are therefore 300 μm separated from each other. The first section of each specimen was aimed at the center of bone defect or slightly proximally and then continued distally on the femur. The bone defect was created at distal end of the femur with a diameter of 2.5 mm and depth of 5 mm (for further information also see section “*Surgical procedure to create and fill femoral bone defects”)*. Three sections of each specimen were stained with methylene blue and basic fuchsine. For the 12-week group, 3 additional sections were made and left unstained for fluorescence analysis and immediately stored in the dark. All sectioning and histological processing was performed at the Radboudumc, department of Biomaterials (Nijmegen, the Netherlands).

#### Descriptive histology and histomorphometrical analysis

At both implantation periods of 4 and 12 weeks, the pMMA histological sections (3 sections per specimen) were examined by light microscopy (Leica Microsystems AG, Wetzlar, Germany); non-stained images were used for fluorescence microscopy. Digital images of the sections were assessed histologically and quantitative histomorphometry was performed using ImageJ computer-based image analysis software (Java® ImageJ 1.47, Image processing and analysis in Java)^71^. For quantitative analysis, a circular region of interest (ROI) with a diameter equal to the created defects (i.e. 2.5 mm) or an extended  ROI (eROI; 3 mm) were superimposed on the defect area. Within the ROI and eROI, the amounts of newly formed bone and material remnants were measured using a combination of color discrimination (staining) and morphological appearance (for verification).

### Statistical analysis

Data are presented as mean with SD for characterization, bone dynamics by fluorochorme labeling and histomorphometrical data. Statistical analyses were performed using STATISTICA 7.0 (StatSoft, Tulsa, Oklahoma, USA) or GraphPad Software (PRISM; La Jolla CA, USA). Shapiro-Wilk normality test was used to check distribution. A Kruskal-Wallis test and Dunn post hoc were used for nonparametric data. One-way analysis of variance (ANOVA) and Tukey multiple comparisons post-tests were used for parametric data. Student’s t-tests were used for comparison of histomorphometrical data between time points for selected materials and paired t-tests were used for comparison between the two composites. Values of p < 0.05 were considered statistically significant.
